# A new flexible plug and play scheme for modeling, simulating, and predicting gastric emptying

**DOI:** 10.1186/1742-4682-11-28

**Published:** 2014-06-10

**Authors:** Shaji Krishnan, Mark van Avesaat, Freddy J Troost, Henk FJ Hendriks, Albert A de Graaf

**Affiliations:** 1 Microbiology and Systems Biology, TNO, Utrechtseweg 48, P.O. Box 360, 3700 AJ Zeist, The Netherlands; 2 Kinetics Research for Food & Pharma, TNO, Utrechtseweg 48, P.O.Box 360, 3700 AJ Zeist, The Netherlands; 3 Department of Internal Medicine, Division of Gastroenterology-Hepatology, University Medical Centre, P. Debyelaan 25, P.O.Box 5800, 6202 AZ Maastricht, The Netherlands; 4 Top Institute Food and Nutrition, Nieuwe Kanaal 9A, 6709 PA Wageningen, The Netherlands

**Keywords:** Modeling, Gastric emptying, Functional modules, Feedback loop

## Abstract

**Background:**

In-silico models that attempt to capture and describe the physiological behavior of biological organisms, including humans, are intrinsically complex and time consuming to build and simulate in a computing environment. The level of detail of description incorporated in the model depends on the knowledge of the system’s behavior at that level. This knowledge is gathered from the literature and/or improved by knowledge obtained from new experiments. Thus model development is an iterative developmental procedure. The objective of this paper is to describe a new plug and play scheme that offers increased flexibility and ease-of-use for modeling and simulating physiological behavior of biological organisms.

**Methods:**

This scheme requires the modeler (user) first to supply the structure of the interacting components and experimental data in a tabular format. The behavior of the components described in a mathematical form, also provided by the modeler, is externally linked during simulation. The advantage of the plug and play scheme for modeling is that it requires less programming effort and can be quickly adapted to newer modeling requirements while also paving the way for dynamic model building.

**Results:**

As an illustration, the paper models the dynamics of gastric emptying behavior experienced by humans. The flexibility to adapt the model to predict the gastric emptying behavior under varying types of nutrient infusion in the intestine (ileum) is demonstrated. The predictions were verified with a human intervention study. The error in predicting the half emptying time was found to be less than 6%.

**Conclusions:**

A new plug-and-play scheme for biological systems modeling was developed that allows changes to the modeled structure and behavior with reduced programming effort, by abstracting the biological system into a network of smaller sub-systems with independent behavior. In the new scheme, the modeling and simulation becomes an automatic machine readable and executable task.

## Introduction

Biological systems analysis with a set of hypotheses at hand is a cyclic process that starts with an experimental design, data acquisition, data analysis, data or hypothesis driven modeling, simulation, and analysis [1,2]. At every cycle, (part of) the description of the biological system is refined either to improve or readdress the hypothesis. This implies that in biological systems analysis, the data/hypothesis driven model is constantly undergoing changes.

Most systems biology modeling tools require the user to manually instruct the computer via the supported programming tools to achieve the modeling and simulation goals [3-5]. Such a task involves programmatically describing the biological components, associated transfer functions and the interactive behavior among the components. There are a few modern systems biology modeling tools like Simbiology [6] and PhysioDesigner [7] that provide the user with graphical supplements to pick commonly used biologically relevant components and connectors from the tool pallet and place them within the model building environment. Nevertheless, the functional descriptions of all the components and interactions between them still need to be programatically described. Thus, a full iterative systems biology modeling cycle in practice often becomes an extremely daunting task. A large scale simplification in modeling can be achieved if programming the functional behavior of a component can be avoided and the task be replaced by integrating sub-units of preprogrammed transfer functional elements.

Every sub-physiological entity such as an organ, or a tissue can be considered as having a well-specified functional behavior defined with respect to its inputs and outputs. The behavior of a biological system is the integrated behavior of these sub-physiological entities working in unison. Thus from a physiological perspective, integrating sub-units of preprogrammed transfer functional elements to realize the functionality of a biological component or biological system as a whole, is apparently relevant.

This paper describes an environment suitable for biological systems modeling and simulation that relieves the re-programming effort usually associated with changes in experimental design and modeling. To demonstrate the working of the proposed modeling and simulation environment and its flexibility to accommodate experimental changes, the gastric emptying behavior observed in humans was modeled. The regulation of gastric emptying forms a key part in the complex process of food intake regulation that is an active area of research [8-10]. Different cell types, hormones, receptors and neural signals all act simultaneously in this system. It is currently largely unclear how signals arising from different parts in the intestine act together in a feedback fashion via the central nervous system to regulate the meal intake behavior. The proposed modeling approach could be of help for allowing researchers to rapidly and easily construct model variants and decide which one offers the most consistent interpretation of experimental data. Therefore, a study aimed at influencing gastric emptying by intestinal infusion of nutrients was chosen for a proof-of-concept example. The parameters of the model estimated from experimental data collected from a control group of subjects were used to predict the gastric emptying rate for an intervention group that received ileal nutrient infusion.

## Design and software environment

**Figure 1:**
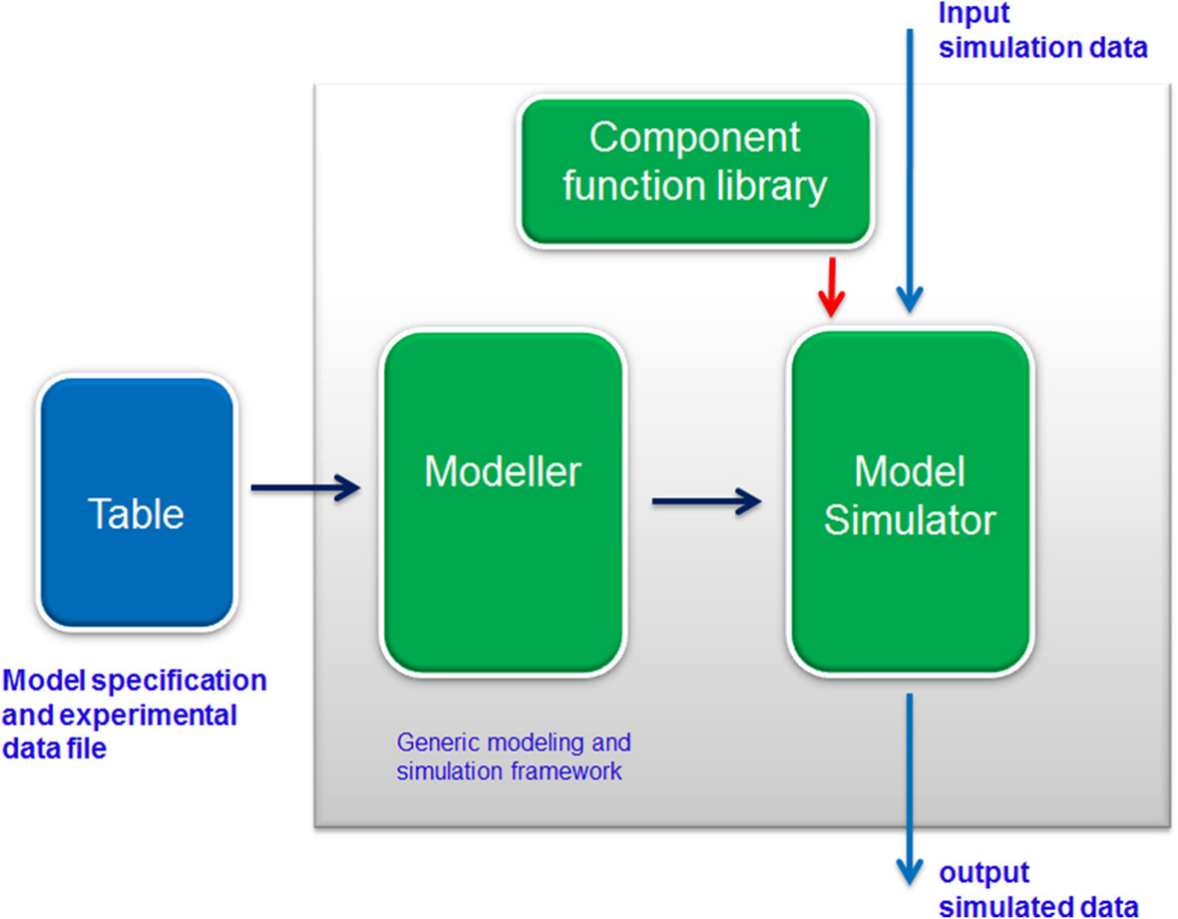
Generic modeling and simulation framework.

From a biological system modeler’s perspective who wants a relief from the re-programming efforts associated with experimental and modeling changes over time, the modeling and simulation environment must allow the user to specify the sub-physiological entities that take part in the biological system modeled along with their input/out relationships in any simple and easily modifiable format. The user should also be capable of providing the modeling and simulation environment with any experimental data collected or supplied at the system level or sub-physiological entities levels. Also, given a model specification and associated experimental data e.g. as input in a textual format, the modeling and simulation environment should automatically construct the model and simulate the modeled behavior.A software architecture able to meet the requirements specified above is illustrated in Figure 1. Central to this architecture is the generic modeling and simulation framework that comprises a model builder, a model simulator, and a component function library. The model builder and simulator are precompiled executables. The simulator dynamically loads the component function library during the simulation run. The user supplies the model specification and experimental data to the generic modeling and simulation framework via a model specification and data file in a predefined format. The model builder parses the model specification file and constructs a model as specified by the user. The simulator loads the constructed model and in conjunction with the component function library simulates the modeled behavior with appropriate simulation data. The following subsections will provide detailed descriptions of the model builder, the model simulator, the component function library, and model specification and data file.

### Model builder

A biological system for the purpose of modeling can be considered an assembly of independent sub-physiological entities that work in unison to achieve certain biological objectives. To model the behavior of such a biological system, it is convenient to choose an abstraction that represents each sub-physiological entity as an independent component that together with other components form a network of components. Such a network, used to model a system is then a component based system model.

The basic unit of a component based system model is a component with a certain number of inputs and outputs. These inputs and outputs are related by a mathematical function. The *structural specification* of a component is thus defined as the name of the component together with the name of its inputs and outputs, while the *functional specification* of a component is defined as the mathematical relationship between its inputs and outputs. The function of the model builder is to construct a component based systems model given the structural and functional specification of the components constituting the modeled biological system.

### Model simulator

**Figure 2:**
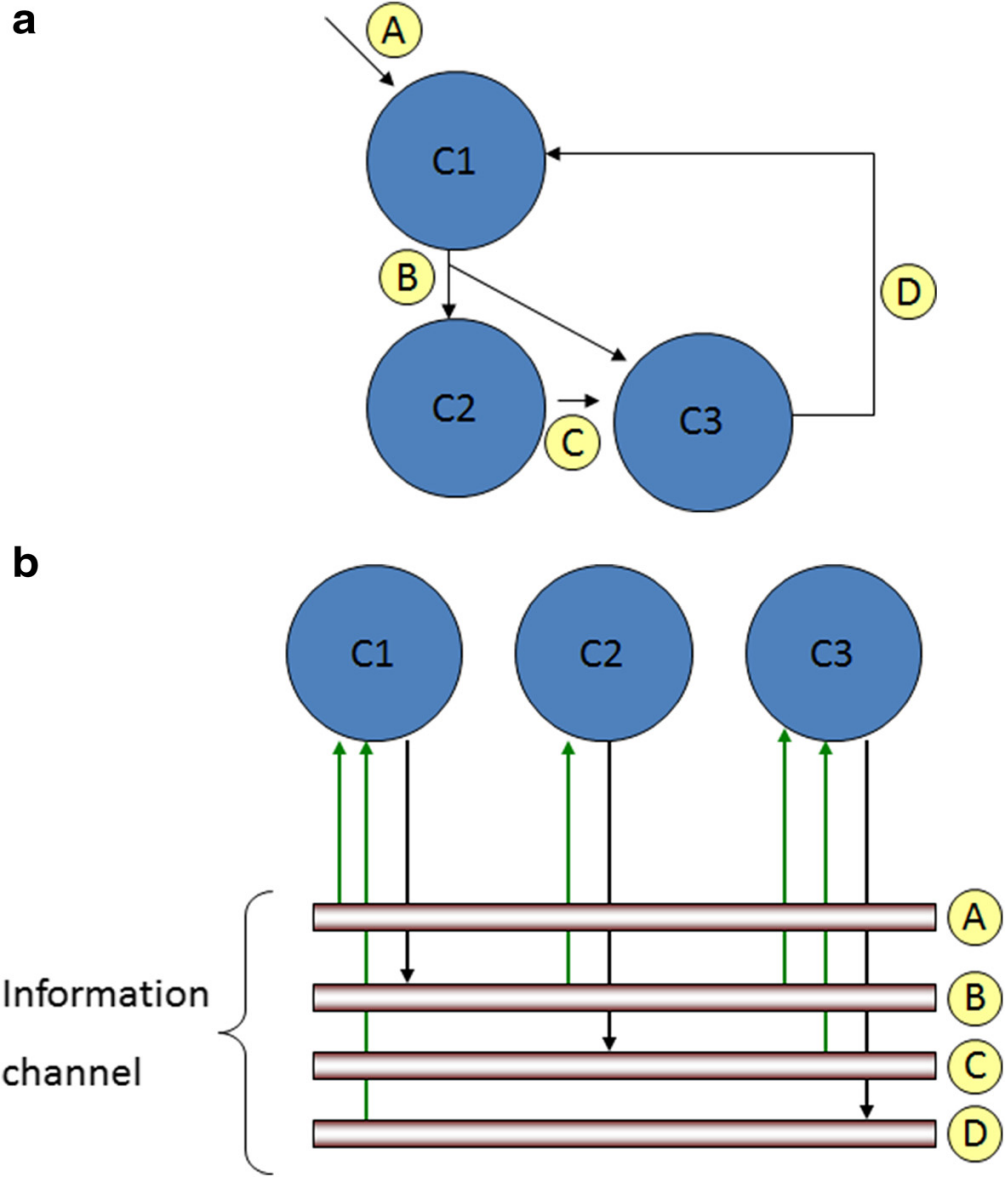
**Example system model. (a)** Structural model description of the example system model. **(b)** Analogous visual representation of the structural model.

The model simulator simulates the component based system model for a predefined number of simulation cycles. A component system model with a set of inputs is said to be simulated for a predefined number of simulation cycles if every component output is evaluated at each simulation cycle. A given simulation cycle is said to be completed if every component outputs have been evaluated for that simulation cycle.The Model builder constructs a component based system model in such a way that any addition or deletion of components, if necessary, is always possible at the completion of a simulation cycle. To illustrate this construction a hypothetical component based system model with 3 components, namely C1, C2, and C3, and the respective interconnections A, B, C, and D among the components is shown in Figure 2a. An other visual representation of the same structural model is depicted in Figure 2b. The two visually represented system models are not different from each other except that in the latter the edges (interconnections) connecting the components are represented as information channels and every component is connected to one of more of the information channels. This representation intuitively matches the physiological situation of organs connected by blood vessels and/or nerve channels. At every simulation cycle the data currently available on the information channel is either read to the inputs of the components (connected in the current simulation cycle) or written to the information channel from the outputs of the components (currently available). The data will be read or written only by those components connected to the information channel in the current simulation cycle. This model construction and simulation feature allows any number of model components to be added to or deleted from the system model during simulation with appropriate control structures.

### Component function library

The component function library contains the functional specification (i.e., the mathematical relationship between the inputs and the outputs) of every component constituting the component system model. Since the simulator has been programmed to simulate the model in time, the functional specification of the components are described as functions of time as well. Functional specification of the components must be defined by the user and updated to the component function library.

### Model specification and experimental data file

The model specification and the experimental data file provided by the user contains two sets of information. The first is the structural specification of the components constituting the systems model and the second is the experimental data relating to experiments performed on the system. The name of the components and respective inputs and outputs are row-wise tabulated. The name of an output of a component is the same as the input of another component if the two are connected and is different if they are not connected. An additional column, “Connect”, is present and has a value either “Yes” or “No”, that connects or disconnects the input/outputs of the respective components. This column is added to introduce an additional flexibility to associate or disassociate the respective connection between components.

For the hypothetical system model depicted earlier in Figure 2a, the components, C1, C2, and C3, are row-wise tabulated in Table 1. The input of the component, C1, is, A and D, and the output is, B, which then is the input to the component, C2. The descriptions for the other components are similar. Note that the input C from component C3 has been disconnected by entering “No” in the “Connect” column. The experimental data is time-wise tabulated at the row for each input and the output of the component. For example, the input A to the component C1 at time 0, is 20 units and remains zero for the rest of the time (5-30). The entries are blank if experimental data are not available.

**Table 1:** **Example of model specification file for the structural model description of the model in Figure **2**a**

**Components**	**Connect**	**Inputs**	**Outputs**	**0**	**5**	**10**	**15**	**20**	**25**	**30**	**35**
C1	Yes	A		20	0	0	0	0	0	0	0
	Yes	D									
	Yes		B								
C2	Yes	B									
	Yes		C								
C3	Yes	B									
	No	C									
	Yes		D								

## Results

Gastric emptying, along with intestinal motility, secretion of digestive enzymes and peptide hormones are important physiological processes involved in the regulation of the meal digestion process [11,12]. Gastric emptying is a physiological process in which the stomach will gradually empty its content into the small intestine. The content will then stimulate the release of several hormones (CCK, PYY, GLP-1 etc.) by the intestinal mucosa, which elicit feedback signals through various neural pathways. One of these neural pathways acts as a feedback to the gastric emptying process itself. The vagal afferent path commences from the gut and terminates at the Nucleus Tractus Solitarius (NTS) of the central nervous system [13]. The response or the negative feedback arises from the central nervous system via the vagal efferents and terminates at locations including the stomach, slowing down the emptying rate of the stomach [14].

In several studies it has been shown that ileal infusion of nutrients results in a delay in gastric emptying and small bowel transit time, and an enhanced release of gastrointestinal hormones. Investigating the mechanism of this so-called ileal brake activation is of potential interest for the development of functional foods that release nutrients in the distal part of the small intestine. Furthermore, Maljaars et al. [12] showed that ileal infusion of lipid (safflower oil) resulted in a more potent intestinal brake effect when compared to duodenual infusion. Gastric emptying was significantly delayed in ileal infusion compared to duodenal infusion (206 min vs. 138 min) [12]. Numerous models have been reported in literature capable to simulate or predict the gastric emptying rate in humans [14-16]. However, in most of these models only the stomach and the intestine have been considered as the participating components [17]. The full feedback loop of the gastric emptying process i.e. involving gradual release of the nutrients from the stomach and subsequent release of hormones that elicit neural signals from the gastrointestinal tract that effect further release of food from the stomach (and also intake of new food) in a feedback scheme via the central nervous system have not been comprehensively taken into consideration. Apart from this, the modeling and simulation schemes, as reported in these publications, involve rigorous re-programming steps in case the experiment needs to be re-designed.

In order to illustrate the component based modeling process within the proposed modeling and simulation environment, the following sections will discuss the gastric emptying modeling and simulating process with a minimal set of components. The predictive capability of the constructed systems model will then be investigated with appropriate experiments conducted on human volunteers.

### Modeling gastric emptying behavior

To construct a system level gastric emptying model, the structural specification of all components that constitute the model along with the experimental data will be described in the model specification and data file. The functional specification of the components will then be added to the component function library. The constructed gastric emptying model together with the component function library and the experimental data specified in the model specification and the data file will be simulated to estimate the parameters of the model. In practice the model is used to answer a particular research question. i.e., “How does nutrient X influences gastric emptying rate Y?”

#### Structural specification

**Figure 3:**
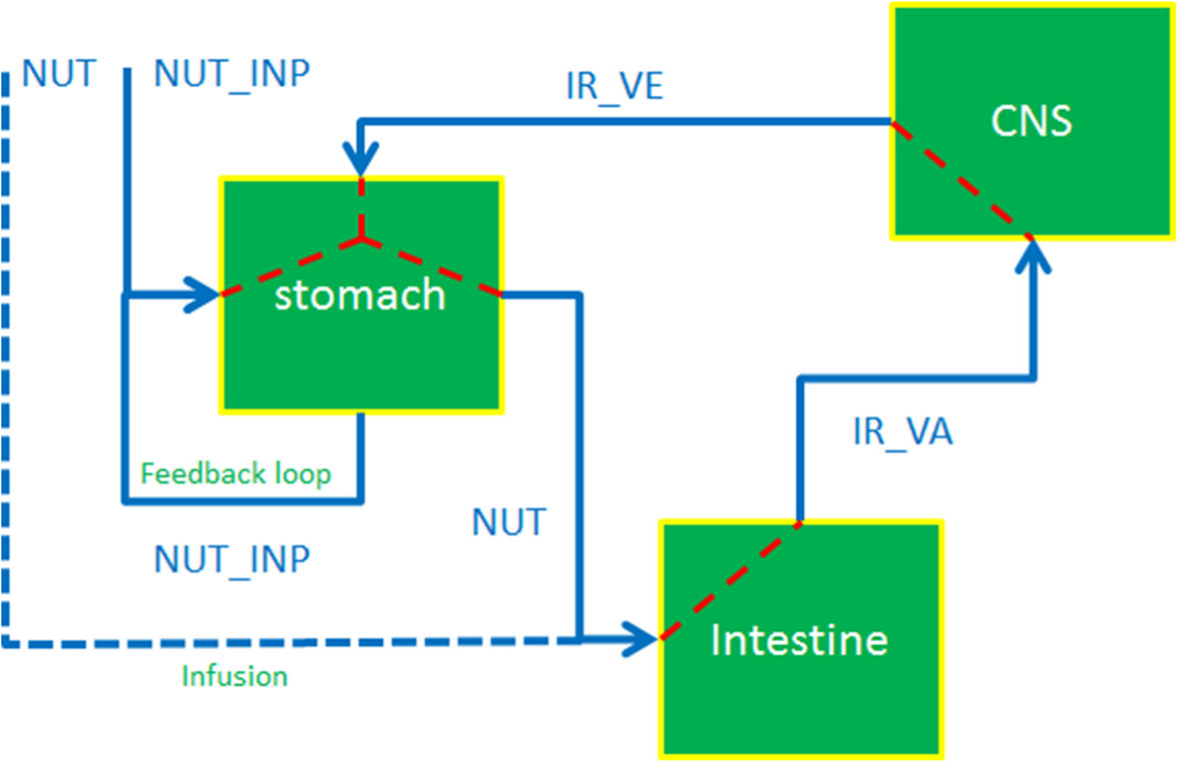
Diagrammatic representation of the structural model for the gastric emptying example.

Table 2 shows the content of the structural specification and data file for the gastric emptying model. A diagrammatic representation of the structural model is shown in Figure 3. The components that constitute the structural model are Stomach, Intestine (GI), and Central Nervous System (CNS). NUT_INP (Nutrient Input), is the input to the component Stomach. The other input, IR_VE (Intestinal Response - Vagal Efferents), is the feedback from the the CNS. The reason why the output and the input of the stomach are combined and commonly referred as NUT_INP will become clear when the functional model of the Stomach is described. The other output of Stomach, NUT (Nutrient) is the input to the next component Intestine. An external input NUT tied to the input of the Intestine is an infusion input that can modulate the gastric emptying phenomena. In the experimental setting, this infusion is administered via a catheter inserted in the GastroIntestinal (GI) tract, with the catheter tip positioned in the distal small intestine (the ileum). The output of Intestine, IR_VA (Intestinal Response - Vagal Afferents) is the input to the next component CNS. The output of the CNS, IR_VE, as explained earlier, is the feedback to the component Stomach.

**Table 2:** Content of a structural specification and data file for the example gastric emptying model

**Components**	**Connect**	**Inputs**	**Outputs**	**0**	**5**	**10**	**…**	**30**	**…**	**120**	**…**	**240**
Stomach	Yes	NUT_INP		35.6								
	Yes	IR_VE										
	Yes		NUT									
	Yes		NUT_INP									
Intestine	Yes	NUT						0.5	…	0.5		
	Yes		IR_VA									
CNS	Yes	IR_VA										
	Yes		IR_VE									
	Yes		VAS									

The experimental data segment of the structural model file contains data for each time point which either are the external input values to the system model or experimentally measured values at the outputs of the components that constitute the system. In the gastric model example external input is supplied at NUT_INP in form of a standardized breakfast meal [18] at time ‘0’ mins (expressed as the caloric value of the standardized breakfast), and the infusion external input NUT at a time ‘30’ mins until ‘120’ mins with steps of 5 minutes (expressed as the caloric value supplied per 5 min). The rest of the input/output values for all components between time ‘0’ and ‘240’ with time step of ‘5’ mins were either not measured or not present and hence left blank.

#### Functional specification

The dynamics of gastric emptying is functionally described in the component stomach. The intestinal feedback regulating the gastric emptying is functionally implemented as a brake mechanism that slows the gastric emptying rate constant. For the components intestine and CNS, instead of a detail physiological model, a grey-box model with minimal functional elements and associated parameters were chosen. Functional model descriptions for each component constituting the gastric emptying model are described in the following sub-sections and the associated parameters to be estimated during model calibration are shown in Table 3.

**Table 3:** Parameter definitions

**Parameter name**	**Parameter**	**Unit**	**Value**
Gastric emptying rate constant	k	*min*^−1^	To be estimated
Efferent signal threshold	THD	dimensionless	To be estimated
IR transfer rate constant	IR_TR	*E*^−1^	To be estimated
In-vivo decay rate constant	INV_DR	*min*^−1^	To be estimated
Caloric grade	CAL_GRD	dimensionless	0.6
Time at maximum amplitude	T_MAX	min	10
Transfer constant	TRF_K	dimensionless	1
Break constant percentage	BRK	dimensionless	3

##### *Stomach*

The component stomach has two inputs: *NUT*_*INP* and *IR*_*VE*, two outputs: *NUT* and *NUT*_*INP*. For an initial caloric input, *NUT*_*INP*(0), the caloric input *NUT*_*INP*(*t*) retained by the stomach at a time *t* is described by Equation 1, where t is the time in minutes, k is the gastric emptying rate constant per minute and b is the extrapolated y-intercept from the terminal portion of the emptying curve [15].

(1) $$\text{NUT\_INP}(t)=\text{NUT\_INP}(0)*\left(1-{\left(1-e^{-k*t}\right)}^{b}\right)$$

Rewriting Equation 1 in the difference equation form results in Equation 2, where *NUT*(*t*+*Δt*) is the calories expelled from the stomach to the intestine at *t*+*Δt* and *Δt* is the simulation interval.

(2) NUT(t+Δt)=NUT_INP(t+Δt)−NUT_INP(t)=f(t)∗Δt∗CAL_GRD,

where $f(t)=\text{NUT\_INP}(0)*b*k*{\left(1-e^{-k*t}\right)}^{b-1}*e^{k*t}$, and CAL_GRD is the caloric grade value defined as the percentage caloric input absorbed by the Intestine. Assuming an equal distribution and absorption of calories along the intestine, the proportion of calories absorbed by ileum can be approximated by the percentage surface area of ileum. The total length of duodenum, jejunum, and ileum is 25, 260, 395 cm [19]. Assuming a constant radius of the intestine, the percentage surface area of ileum is 60% and thus a value of 0.6 was chosen for CAL_GRD [20].

In the model, the gastric emptying rate constant k is reduced by a percentage BRK, upon each instance that the intestinal response efferent transfer function *sgmd* exceeds a fixed threshold constant THD (Efferent signal threshold constant). The intestinal response efferent transfer function *sgmd* is defined by Equation 3. The value of b in Equation 1 is then calculated as *b*=*e*^*k*∗*T*_*L* *A* *G*^ from k given the value *T*_*LAG*, the initial delay in gastric emptying [15].

(3) $$\text{sgmd}(t)=2/(1+e^{-\text{IR\_TR}*\text{IR\_VE}(t)})-1,$$

where *IR*_*TR* is the intestinal transfer rate constant, and *IR*_*VE* is the intestinal vagal efferent response from CNS.

To find a suitable value for BRK, we considered that the model is evaluated with a time resolution of 1 minute, allowing the system to execute at maximum 1 break event per minute. Furthermore, for the range of nutrient-induced variation in stomach emptying half time we took as a reference data from Robertson et al. [21], showing that adding n-6 polyunsaturated fatty acids (PUFA) vs. n-3 PUFA to a meal can result in an increase of stomach half-emptying time from 155 to 237 minutes. We then required that 15 consecutive break events be sufficient to increase Thalf from 155 to 237 minutes, so as to allow a significant reduction of the stomach emptying rate well within the length of the infusion period of 90 minutes employed in the experiment. This resulted in a value of 0.03 or 3% for BRK (i.e. 155∗1.03^15^≈237).

##### *Intestine*

The component intestine has an input: *NUT* and an output *IR*_*VA*. The intestinal vagal afferent response *IR*_*VA*(*t*+*Δt*) at time t is the convoluted vagal afferent response in E (arbitrarily chosen) units to the intestinal caloric input from 0 until t as shown in Equation 4.

(4) $$\text{IR\_VA}(t+\mathrm{\Delta t})=\overset{t/\mathrm{\Delta t}}{\underset{i=0}{\sum }}a*{\left(t-(\mathrm{\Delta t}*i)\right)}^{b}*e^{-c*(t-(\mathrm{\Delta t}*i))}$$

where *a*=*NUT*(*Δt*∗*i*)∗(*c*∗*e*/*b*)^*b*^, the in-vivo (intestinal vagal afferent response) decay rate constant, *c*=*INV*_*DR*, and *b*=*T*_*MAX*∗*c*, where T_MAX is the time at which the intestinal vagal afferent response to the intestinal input is maximum. To choose a value for T_MAX we took the time to maximum response of the hormone most closely associated with regulation of the stomach emptying rate, i.e. CCK, as a reference. This time was read from Figure one A in [22] as 10 minutes.

##### *CNS*

The component CNS has an input: *IR*_*VA* and an output *IR*_*VE*. The efferent response of the CNS, *IR*_*VE* to the afferent input *IR*_*VA* is defined in Equation 5.

(5) IR_VE(t+Δt)=TRF_K∗IR_VA(t)

Since we were unable to find quantitative data on gut-brain afferent-to-efferent neural signal transduction, we assumed a direct proportional unit transfer (TRF_K = 1) for simplicity reasons.

### Gastric emptying protocol and model calibration

The experimental protocol for calibrating, and predicting the gastric emptying model followed the main principles described in [23] with minor modifications. At time t = 0 mins, a standard solid meal was consumed by the volunteer^a^. ^13^C octanoic acid was added to the standard breakfast meal to measure gastric emptying rate. Although ${}^{13}{\text{CO}}_{2}$ breath test does not directly measure the gastric emptying, it has been shown to correlate well with the gold standard scintigraphy in several studies. However, none of the various mathematical models used to extract Thalf values from the measured 13C enrichment data was shown to be universally suited for all the different applications of the test. For an in-depth discussion, the reader is referred to [24]. The methodology is based on the firm retention of ^13^C-octanoic acid in the solid phase of a standard test meal during its passage through the gastric environment, followed by a rapid disintegration of the solid phase in the duodenum with subsequent absorption of ^13^C octanoic acid and hepatic oxidation to ${}^{13}{\text{CO}}_{2}$, which is exhaled in breath. It has been shown that the post-gastric metabolism (absorption of ^13^C octanoic acid, hepatic metabolism to ${}^{13}{\text{CO}}_{2}$ and excretion via breath) are similar, thus less influential, between individuals [16]

At t = 30 mins, a solution containing either saline (placebo) or safflower oil (SO) was infused into the ileum. The perfusion was performed with an pump connected to the nasoileal tube. The infusion continued for a period of 90 minutes (i.e. until t = 120 mins) at a rate of 1 mL/min. The breath samples were taken at the following time points; 15 minutes before the meal and at 15, 30, 45, 60, 75, 90, 105, 120, 180, 210 and 240 minutes after the standard breakfast meal. From each of the breath samples the percentage dose/h of ^13^C exhaled were measured. The Thalf and TLag were computed from the percentage dose/h of ^13^C measurements [25,26].

When inspecting the ^13^C breath test data we were confronted with large inter-and intra- individual variation of the Thalf values estimated from the ^13^C enrichment values. For this reason we refrained from paired test analysis but rather took a population-based approach. We undertook a model predictive capability test using 3 different selections of the ^13^C data, as follows, S1: the complete data set; S2: the data set from which all curves that showed one or more instances of occurrence of a negative ^13^C enrichment value had been discarded; S3: the dataset from which all curves classified as outlier based on the Chi-squared criterion were discarded. A measured value was classified as an outlier if the Chi-squared score ($\chi_{i}^{2}={\left(x_{i}-\bar{x}\right)}^{2}/s^{2}$), where *x*_*i*_ is the mean of the ^13^C breath test measurements for the *i*^*t* *h*^ subject, $\bar{x}$ is the overall mean of the ^13^C breath test measurements, and s is the standard deviation, was greater than 1. Selection S1 is the most complete, but has the drawback that the large inter-individual variation can obscure the treatment effect thus reducing the significance that can be associated with the model predictive capability testing. Selection S2 should suffer less from this problem, while retaining more of the data. Selection S3 can be considered the most stringent for our model testing purpose. Therefore, we concentrate on the results obtained with data selection S3 and bring results with data selections S1 and S2 only for comparison.

**Figure 4:**
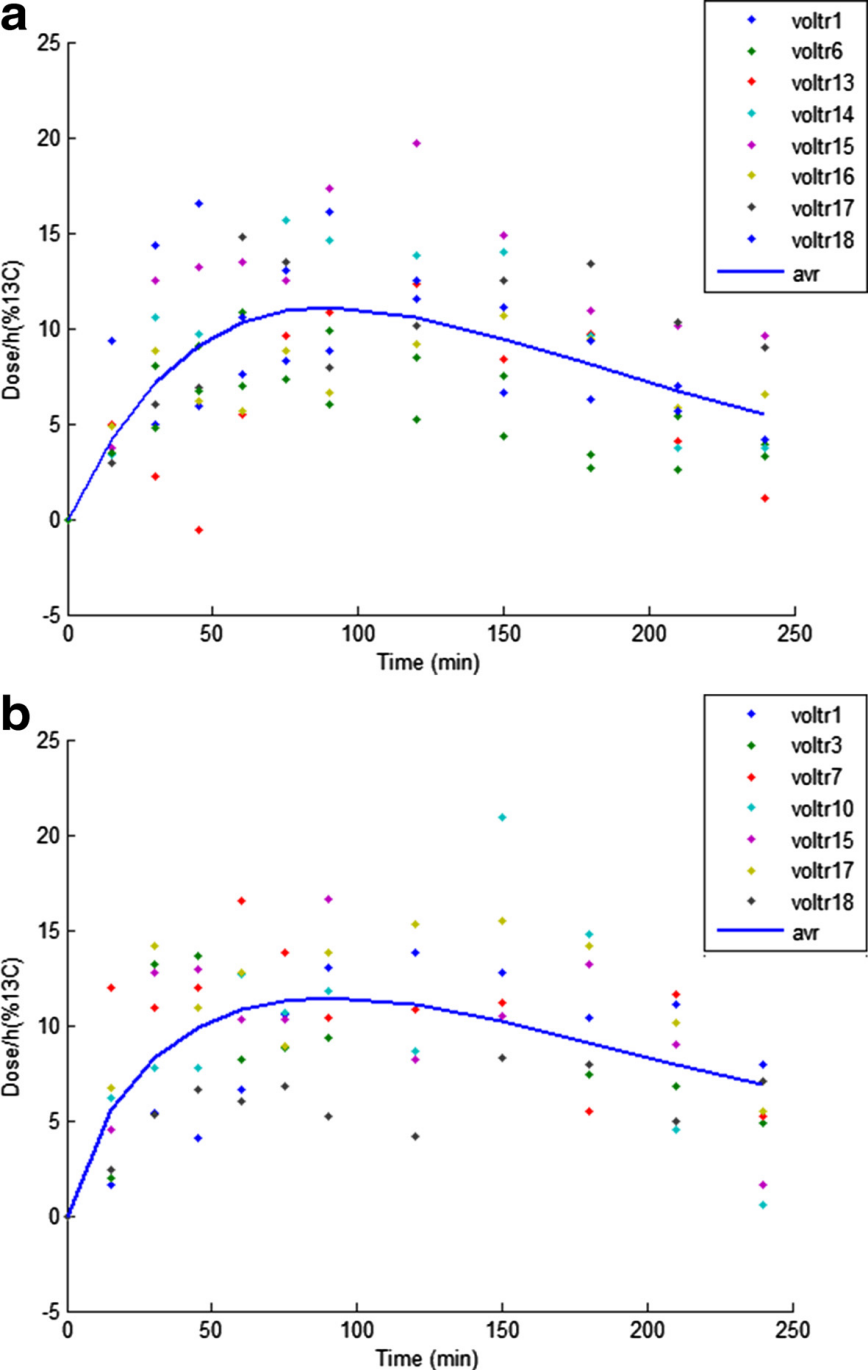
**Accumulated %**^**13**^**C measurements (Dose/h) for (a) Placebo infusion, (b) Safflower infusion, and the non-linear least squares fit (continuous line) of a curve of the form *y=at***^***b***^ ***e***^***−ct***^**.**

In the calibration step, the gastric emptying model parameters shown in Table 3 were estimated. For placebo data selection S3, the ^13^C measurements (Dose/h[% ^13^C]) from the volunteers 1, 6, 13, 14, 15, 16, 17, and 18 (data available as a supplement to the manuscript Additional file 1) corresponding to the placebo infusion were chosen to estimate the % ^13^C curve constants (a, b, c; *y*=*at*^*b*^*e*^−*c* *t*^[25]) from which the half emptying time (*THalf*_*P* *B*_), and lag time (*TLag*_*P* *B*_) for placebo infusion were calculated by fitting a single curve to all the data (population model). The gastric empyting model parameters were then estimated by simulating the model with experimental input conditions corresponding to the placebo infusion and optimized using a non-linear least squares fitting procedure, for the parameters that result in a gastric emptying curve with a half emptying time, and lag time equal to *THalf*_*P* *B*_ and *TLag*_*P* *B*_, respectively. The % ^13^C curve that best fitted the set of ^13^C measurements (Dose/h[% ^13^C]) from the volunteers is shown in Figure 4a. The % ^13^C curve constants estimated from the least square fit were a = 0.289, b = 1.05, and c = 0.011. The calculated *THalf*_*P* *B*_, and *TLag*_*P* *B*_ for placebo infusion using these constants were 150.93 min, and 91.72 mins, respectively. The gastric empyting model parameters then estimated by simulating and optimising from the half emptying time and lag time for the placebo infusion were k = 0.009, THD = 0.45, IR_TR = 0.3, and INV_DR = 0.1.

### Prediction

**Figure 5:**
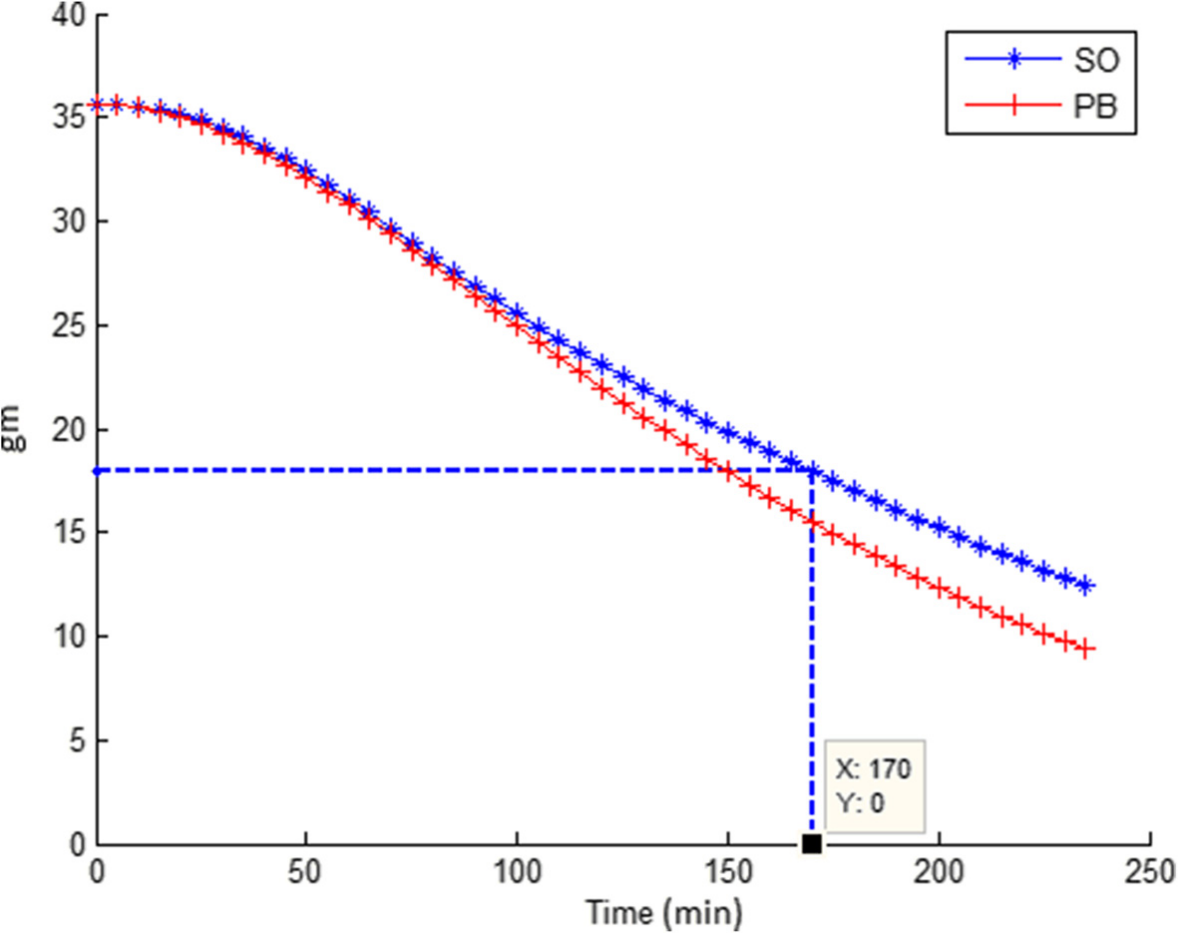
**Gastric emptying curves for safflower oil infusion (SO) and placebo infusion (PB).** The former was predicted based on a model parameter fit of data from the placebo infusion experiment.

The gastric emptying model with the parameters estimated was used to predict the half emptying time and lag time for the safflower infusion. The experimental input conditions for the safflower infusion were simulated and the resulting half emptying time *THalf*_*S* *O*_ and lag time *TLag*_*S* *O*_ for the safflower infusion was determined to be 170 mins, and 91.72 mins respectively (see Figure 5). The result was then compared with the *THalf*_*S* *O*_, and *TLag*_*S* *O*_ calculated from gastric emptying curve fit parameters determined from the set of ^13^C measurements (Dose/h[% ^13^C]) from the volunteers corresponding to the safflower infusion.

For safflower oil data selection S3, the set of % ^13^C measurements (Dose/h[% ^13^C]) from the volunteers 1, 3, 7, 10, 15, 17 and, 18 (data available as a supplement to the manuscript Additional file 1, volunteer: 16 with a Chi-squared score 0.94 is expectionaly treated as outlier for 2 reasons: 1. The maximum % ^13^C measurement was lower compared to the rest of the good measurements, 2. % ^13^C when treated separately, resulted in a Thalf value and Tlag of 401.74 mins and 95.58 mins, respectively, which were values least likely to be a physiologicaly plausible gastric emptying half time, and lag time, for the given test meal intake) who had safflower infusion were chosen to estimate the % ^13^C curve constants (a, b, c). The curve that best fitted the set of % ^13^C measurements (Dose/h[% ^13^C]) from the volunteers is shown in Figure 4b. From the estimated % ^13^C curve fit parameters, the calculated experimental *THalf*_*S* *O*_, and *TLag*_*S* *O*_ for safflower oil infusion were 168.03 mins and 94.07 mins, respectively. The error in gastric emptying model prediction was thus found to be less than +2 mins.

The model was also re-run by classifying the data as full data (S1), and partial data (S2; curves with 1 or more negative ^13^C enrichment values discarded). The results are shown in Table 4. The predicted THalf error stayed within 6% of the measured value which seemed acceptable given the amount of variation in the measured data.

**Table 4:** **Gastric emptying experimental data (measured) and model predictions (predicted) for 3 different data selections S1 (all data), S2 (curves with 1 or more negative**^**13**^**C enrichment values discarded) and S3 (data selection based on chi-squared criterion) as described in the results section**

**No.**	**Placebo**	**Safflower oil**	**THalf (min); TLag (min)**	**THalf (min); TLag (min)**
	**(vol. no.)**	**(vol. no.)**	**(measured)**	**(predicted)**
S1	1, 3, 6, 7, 8,	1, 3, 6, 7, 8,	172.8; 103.7	165; 86.5
	10, 12, 13,	10, 12, 13,		
	14, 15, 16,	14, 15, 16,		
	17, & 18	17, & 18		
S2	1, 3, 6, 7,	3, 6, 7, 8,	174; 101.3	165; 87.5
	10, 12, 13,	10, 14, 15,		
	14, 16, 17,	16, 17, & 18		
	& 18			
S3	1, 6, 13, 14,	1, 3, 7, 10,	170; 91.7	168; 94.7
	15, 16, 17,	15, 17, & 18		
	& 18			

## Discussion

A software executable comprising a model builder, a simulator and a dynamically loadable component function library was realized using MATLABⒸ R2012b (32-bit) [27]. This software executable with a user supplied (i) component structural specification and data file and (ii) component functional specification, as input can model a biological system especially suited for physiological modeling. The architecture is flexible to modeling changes either at the structural (sub-physiological entities and their interconnections) or functional (behavioral) level without any re-programming effort. Modeling various functional behaviors of the biological system, one at a time, requires the user only to add corresponding functional behavior of the component in the component functional library and after necessary textual modifications in the structural model and data file, the modeling and simulation environment is ready for simulating the specified behavior. In this sense, the modeling and simulation environment is a plug and play system with no re-programing effort and hence reusable.

The flexibility of the modeling and simulation environment was demonstrated by modeling and simulating the gastric emptying behavior in humans. Not only the stomach, but also the gut and the central nervous system were added to model a feedback mechanism that regulates the stomach emptying. Structurally specifying these sub-physiological entities as additional components was performed without any additional programming effort. Since the model specification and data file was designed in a way that every input and output of the component were directly accessible, the experimental input data associated with the infusion of the nutrients into the gastro-intestinal tract were easily supplied to the model without any modification. Another flexible feature was the choice on the functional specification of the components, especially the gastric emptying function of the stomach. There are several alternative functions to describe the gastric emptying phenomena from the ^13^C measurements [25,26]. The user had the choice to pick the functional description that best fitted the % ^13^C measurements and then provide this function as the functional specification of the stomach to the component function library. The simulator then dynamically loaded the user-provided functional specification of the stomach and ran simulation.

The gastric emptying model constructed by the model builder with the input provided by the model specification and data file was calibrated to estimate the parameters of the model. For calibration purposes the TLag, and Thalf calculated from the average ^13^C measurements with a placebo infusion were used. With the gastric emptying model parameters estimated from the calibration run, the gastric emptying curve with a value for TLag, and Thalf was predicted for a safflower infusion. The error in the predicted results when compared to the measured results was less than 2 minutes for the most stringent data selection S3, showing that indeed the model even in a simple form was able to correctly describe gastric emptying functional behavior. Model performance evaluations based on broader data selections S1 and S2 showed a decreased accuracy that however remained within 6% of the experimentally determined value and therefore can be considered satisfactory in view of the much larger that showed a much larger inter-individual variation in the breath test-derived ^13^C curves.

The sample size even in data selection S1 was not large enough to perform a bootstrapping in the calibration phase and a subsequent cross-validation in the prediction phase. The standard deviation of individual Thalf estimates derived from ^13^C breath test curves of individuals included in the safflower oil group of data selection S3 was determined to be 18.2 minutes. The model prediction of Thalf for the safflower oil experiment was only 2 minutes different from the experimentally determined value for the population, i.e. much less than this standard deviation. Therefore the model predictive capacity is considered very good for this specific case.

The reason for not being able to verify the model performance in the paired test performed in volunteers is the limitation on the quality of the available % ^13^C measurements (strictly based on the statistical test for outliers) as discussed in the Results section. Nevertheless, the model prediction error was calculated for the paired test performed in volunteers 1, 15, 17 and 18, where % ^13^C measurements were available (results available as a supplement to the manuscript Additional file 2).

Obviously, the chosen proof-of-concept study only addressed a very limited part of the complex physiology involved in intestinal feedback signaling to regulate stomach emptying and, in a broader context, food intake. Therefore we cannot from the present study alone draw any conclusions on the general applicability and value of the proposed new plug-and-play modeling scheme. This has to await application to further studies that consider substantially more biological aspects.

Some of the currently available modeling and simulation tools like COPASI [28] and Simbiology [6] are excellent for biological process modeling and simulation but not very much suitable for modeling physiological behavior. The general modeling strategy of these tools is to model the biological system as a network of reactions linking substrates (to products via formation processes, of which the kinetic behavior is described. The reaction rates are either user-formulated or chosen from a set of known kinetic functions such as Michaelis-Menten enzyme kinetics, etc. Depending on the requirement such as determining the steady state solutions, sensitivity analysis, etc., these tools are able to perform the task by solving a set of either differential or stochastic equations. In contrast, the modeling and simulation tool PhysioDesigner [7] that is built on ISML [29] is able to model and simulate physiological processes at the organ level. However, since ISML is structured language it requires the user to programmatically describe the interactions of physiological entities that constitute the modeled biological system.

Table 5 provides a comparative overview of some of the commonly used tools for modeling and simulating biological systems. Given the requirements, the plug-and-play modeling and simulation environment and tool proposed in this paper is functionally similar to PhysioDesigner. However, the methodological aspects of PhysioDesigner with respect to model construction and simulation are less flexible to the actual model development situation encountered in practice, especially when the model development is closely associated to related experimental studies. In cases when model development is closely related to an experimental study that generates sufficiently large amount of data at several physiological levels, the inputs and outputs of every component constituting the modeled biological system should be tightly coupled to the respective inputs and outputs of the represented physiological entity with respect to structure and data. One of the main reasons to comply to this requirement is to lessen the discrepancies between simulated and experimental data and thus faithfully modeling the overall physiological behavior. Thus by tightly coupling the experimental data with the structural specification of the model, the table based model specification and the experimental data file input of the proposed modeling and simulation tool is unique in construction and satisfies the practical requirements of system biological modeling. Additionally, this flexible format of specification and the experimental data file allows structural and data changes to be fed into the model at real time without user intervention thus generating the openings for dynamic model building.

**Table 5:** Benchmarking the newly developed plug-and-play modeling tool against commonly used and established tools

**No.**	**Requirements**	**Proposed tool**	**COPASI/SimBiology**	**PhysioDesigner**
1	Organ level modeling, and simulation	Models and simulates at organ level	Models and simulates at cellular level	Models and simulates at organ level
2	Lesser programing effort for:	Programing effort for:	Programing effort for:	Uses ISML (standard programing language)
				and thus requires programing effort.
	1. Structural description	1. Structural description: No	1. Structural description: No	
	2. Functional description	2. Functional description:	2. Functional description:	
		single time effort, and reusable	No (COPASI, but not reusable); single time effort and reusable (SimBiology)	
	3. Experimental data	3. Experimental data: No	3. Experimental data: No	
3	User interface for model structural description	Textual thus machine readable and adaptable	Graphical	Graphical
	(components and interconnections)			
4	Dynamic* model construction * Adaptive to experimental data collected, and structural change requests in time	Dynamic model construction is possible	Dynamic model construction is not possible	Dynamic model construction is not possible
5	Experimental data suppliable at the level of	Possible without any additional interface	Not possible without additional interface	Not possible without additional interface
	physiological entities constituting the model			

## Conclusion

This paper describes a new plug-and-play scheme for biological systems modeling with a successful a proof-of-concept application. The proposed modeling and simulation software environment allows for a reduced programming effort needed to accommodate changes to the modeled structure. This property is gained by abstracting the biological system into a network of smaller sub-systems or components that all exhibit independent behavior. Once the functional specifications of the individual components have been programmed, the modeling and simulation for an arbitrarily network configuration of these components becomes an automatic machine readable and executable task. Experimental data may be included with the structured input information or can be read from a separate database. As proof-of-concept, the new plug-and-play scheme was used to model human gastric emptying with a minimal set of functional components and to accurately predict the increase in stomach emptying half-time caused by ileal infusion of safflower oil vs. placebo.

## Endnotes

^a^ Volunteers signed a written informed consent prior to participation, the study was conducted according to the principles of the Declaration of Helsinki, the METC azM/UM approved the study.

## Competing interests

The authors declare that they have no competing interests.

## Authors’ contributions

SK conceptualized and programmed the architecture for the generic modeling and simulation framework, built the gastric emptying model, conducted the model analysis and drafted the manuscript. MvA carried out the human experimental study and collected the data. FT and HH conceived of the study, and participated in its design and coordination and helped to draft the manuscript. AdG participated in the development of the gastric emptying model, helped in conducting the model analysis and drafting of the manuscript. All authors read and approved the final manuscript.

## Supplementary Material

Additional file 1 **Gastric emptying related %**^**13**^**C measurements.** Experimental data relating to gastric emptying related % ^13^C measurements in dose/h(Ds./h) and cumulative dose (Cum. ds) units for volunteers for placebo (PB) and safflower oil (SO) infusion are available in Additional file 1 file. Click here for file

Additional file 2 **Model prediction error for paired test.** Calculated model prediction error for the paired test performed in volunteers 1, 15, 17 and 18, where good quality % ^13^C measurements were available. Click here for file
