# Supplementary material for: A new flexible plug and play scheme for modeling, simulating, and predicting gastric emptying
Source: Theor Biol Med Model. 2014 Jun 10;11:28. doi: 10.1186/1742-4682-11-28 (PMC4080776; doi:10.1186/1742-4682-11-28)
Supplement: Additional file 1 — Gastric emptying related % 13 C measurements. Experimental data relating to gastric emptying related % 13C measurements in dose/h(Ds./h) and cumulative dose (Cum. ds) units for volunteers for placebo (PB) and safflower oil (SO) infusion are available in Additional file 1 file. [file 1742-4682-11-28-S1.pdf]

Gastric emptying measurements

S. Krishnan, Mark Avesaat, Freddy Troost, Henk Hendriks, Albert de Graaf

October 4, 2013

Tables 1–5 are the experimentally obtained $\%^{13}C$ values in Dose/h(Ds./h) and cumulative dose (cum. ds) units for volunteers (Voltr.x, where x = 1, 3, 6, 15, 17, 18, 16, 14, 13, 12, 10, 7, 8) at time (T) expressed in minutes (min) for placebo (PB) and safflower oil (SO) infusion.

Table 1: Gastric emptying results

T(min)	Voltr.1				Voltr.3				Voltr.6			
	SO		PB		SO		PB		SO		PB	
	Ds./h	Cum. ds.	Ds./h	Cum. ds.	Ds./h	Cum. ds.	Ds./h	Cum. ds.	Ds./h	Cum. ds.	Ds./h	Cum. ds.
0	0	0	0	0	0	0	0	0	0	0	0	0
15	-0.2	0	3.4	0.4	1.6	0.2	3.5	0.4	2	0.3	4.9	0.6
30	5	0.6	5	1.5	5.4	1.1	4.8	1.5	7.1	1.4	8	2.2
45	3.8	1.7	5.9	2.8	4.1	2.3	6.7	2.9	8.5	3.3	9.1	4.4
60	6.7	3	7.6	4.5	6.6	3.6	7	4.6	14	6.1	10.8	6.8
75	9.5	5	8.3	6.5	10.6	5.7	7.3	6.4	16.5	9.9	13	9.8
90	9.7	7.4	8.8	8.6	13	8.7	6	8.1	36.1	16.5	9.9	12.7
120	10.9	12.6	11.5	13.7	13.8	15.4	5.2	10.9	28.5	32.7	8.5	17.3
150	10.8	18	6.6	18.2	12.8	22	4.3	13.2	34	48.3	7.5	21.3
180	9.8	23.1	6.3	21.5	10.4	27.9	3.4	15.2	21.3	62.1	2.7	23.8
210	6.6	27.2	7	24.8	11.1	33.2	2.6	16.7	21.4	72.8	5.4	25.8
240	7.6	30.8	3.9	27.5	7.9	37.9	3.3	18.1	21.7	83.5	3.9	28.1

Table 2: Gastric emptying results

T(min)	Voltr.15				Voltr.17				Voltr.18			
	SO		PB		SO		PB		SO		PB	
	Ds./h	Cum. ds.	Ds./h	Cum. ds.	Ds./h	Cum. ds.	Ds./h	Cum. ds.	Ds./h	Cum. ds.	Ds./h	Cum. ds.
0	0	0	0	0	0	0	0	0	0	0	0	0
15	6.2	0.8	5	0.6	4.5	0.6	3.4	0.4	6.7	0.8	3.7	0.5
30	7.8	2.5	2.2	1.5	12.8	2.7	10.6	2.2	14.2	3.4	12.5	2.5
45	7.8	4.5	-0.6	1.7	12.9	5.9	9.7	4.7	10.9	6.6	13.2	5.7
60	12.7	7	5.5	2.3	10.3	8.8	7	6.8	12.8	9.6	13.5	9
75	10.7	10	9.6	4.2	10.3	11.4	15.7	9.6	8.9	12.3	12.5	12.3
90	11.8	12.8	10.8	6.8	16.6	14.8	14.6	13.4	13.8	15.1	17.3	16
120	8.6	17.9	12.3	12.5	8.2	21	13.8	20.5	15.3	22.4	19.7	25.3
150	20.9	25.2	8.4	17.7	10.5	25.6	14	27.5	15.5	30.1	14.9	33.9
180	14.8	34.1	9.7	22.2	13.2	31.5	9.6	33.4	14.2	37.5	10.9	40.4
210	4.5	39	4.1	25.7	9	37.1	3.7	36.7	10.1	43.6	10.1	45.6
240	0.6	40.2	1.1	27	1.6	39.8	3.7	38.6	5.5	47.5	9.6	50.6

Table 3: Gastric emptying results

T(min)	Voltr.16				Voltr.14				Voltr.13			
	SO		PB		SO		PB		SO		PB	
	Ds./h	Cum. ds.	Ds./h	Cum. ds.	Ds./h	Cum. ds.	Ds./h	Cum. ds.	Ds./h	Cum. ds.	Ds./h	Cum. ds.
0	0	0	0	0 0		0			0		0	
15	2.4	0.3	4.9	0.6	13.8	1.7	2.9	0.4	-0.2	0	9.3	1.2
30	5.3	1.3	8.8	2.3	15.5	5.4	6	1.5	4.4	0.5	14.3	4.1
45	6.6	2.7	6.2	4.2	19.3	9.7	6.9	3.1	7.5	2	16.5	8
60	6	4.3	5.7	5.7	24.9	15.3	14.8	5.8	-0.9	2.8	10.6	11.4
75	6.8	5.9	8.8	7.5	24.8	21.5	13.5	9.3	6.4	3.5	13	14.3
90	5.2	7.4	6.6	9.4	18.8	26.9	7.9	12	3.6	4.8	16.1	18
120	4.2	9.8	9.2	13.4	17.3	35.9	10.1	16.5	11	8.4	12.5	25.1
150	8.3	12.9	10.7	18.4	27.9	47.2	12.5	22.1	7.7	13.1	11.1	31
180	7.9	16.9	9.4	23.4	26.8	60.9	13.4	28.6	5.5	16.4	9.3	36.1
210	5	20.1	5.8	27.2	10.7	70.3	10.3	34.5	8.5	19.8	5.7	39.9
240	7.1	23.2	6.5	30.3	11.5	75.9	9	39.4	5.6	23.4	4.2	42.4

Table 4: Gastric emptying results

T(min)	Voltr.12				Voltr.10				Voltr.7			
	SO		PB		SO		PB		SO		PB	
	Ds./h	Cum. ds.	Ds./h	Cum. ds.	Ds./h	Cum. ds.	Ds./h	Cum. ds.	Ds./h	Cum. ds.	Ds./h	Cum. ds.
0	0	0	0	0	0	0	0	0	0	0	0	0
15	1.3	0.2	10	1.2	12	1.5	13	1.6	2	0.3	6.3	0.8
30	3.3	0.7	12.2	4	10.9	4.4	20.6	5.8	13.2	2.2	8.7	2.7
45	4.5	1.7	20.1	8.1	12	7.2	20.9	11	13.6	5.5	12.5	5.3
60	7.9	3.3	26	13.8	16.5	10.8	18.7	16	8.2	8.2	12	8.4
75	5.8	5	27.4	20.5	13.8	14.6	16	20.3	8.8	10.4	13.4	11.6
90	5.7	6.4	20.9	26.5	10.4	17.6	11.9	23.8	9.3	12.6	27.7	16.7
120	0	7.8	7.4	33.6	10.8	22.9	14.1	30.3	8.2	17	25.6	30
150	5.9	9.3	12.9	38.7	11.2	28.4	14.7	37.5	8.3	21.1	25.4	42.8
180	8	12.8	12.3	45	5.5	32.6	11.7	44.1	7.4	25	24.2	55.2
210	-2.1	14.3	14.7	51.7	11.6	36.9	13.5	50.4	6.8	28.6	20	66.2
240	-0.7	13.6	4.1	56.4	5.2	41.1	8.2	55.8	4.9	31.5	17.7	75.7

Table 5: Gastric emptying results

T(min)	Voltr.8			
	SO		PB	
	Ds./h	Cum. ds.	Ds./h	Cum. ds.
0	0	0	0	0
15	2.5	0.3	3.8	0.5
30	6.3	1.4	-0.5	0.9
45	8.2	3.2	2.1	1.1
60	8	5.3	3.4	1.8
75	10.9	7.6	0.8	2.3
90	9.6	10.2	0.7	2.5
120	10.7	15.3	-3.1	1.9
150	6.4	19.6	-0.4	1
180	5.5	22.6	-4.3	-0.2
210	5.7	25.4	-3.1	-2
240	4.4	27.9	-4.4	-3.9
